# Supplementary material for: GII.17 norovirus re-emerged in the 2020s as a result of dynamic and adaptive evolutionary processes
Source: Nat Commun. 2025 Nov 24;16:11596. doi: 10.1038/s41467-025-66279-6 (PMC12749941; doi:10.1038/s41467-025-66279-6)
Supplement: Supplementary file 1 — Supplementary Information [file 41467_2025_66279_MOESM1_ESM.pdf]

Supplementary Information

GII.17 norovirus re-emerged in the 2020s as a result of dynamic and adaptive evolutionary processes

Kentaro Tohma[1], Sonja Jacobsen[2], Britta Altmann[2], Joseph A. Kendra[1], Michael Landivar[1], William E. De La O[1], Maria Dolores Fernandez-Garcia[3, 4], Karina A. Gomes[5], Sophia Chudnovsky[1], Lauren A. Ford-Siltz[1], Kelsey A. Pilewski[1], Yamei Gao[1], Ilya Mazo[6], Sandra Niendorf[2], Gabriel I. Parra[1]

1. Division of Viral Products, Center for Biologics Evaluation and Research, Food and Drug Administration, Silver Spring, MD, USA.
2. Department of Infectious Diseases, Robert Koch Institute, Berlin, Germany.
3. Enterovirus and Viral Gastroenteritis Unit, National Centre for Microbiology, Instituto de Salud Carlos III, Madrid, Spain.
4. CIBER Epidemiology and Public Health (CIBERESP), Madrid, Spain.
5. Laboratory of Viral Gastroenteritis, INEI-ANLIS "Dr. Carlos G. Malbrán", Buenos Aires, Argentina.
6. FDA HIVE, Center for Biologics Evaluation and Research, Food and Drug Administration, Silver Spring, MD, USA.

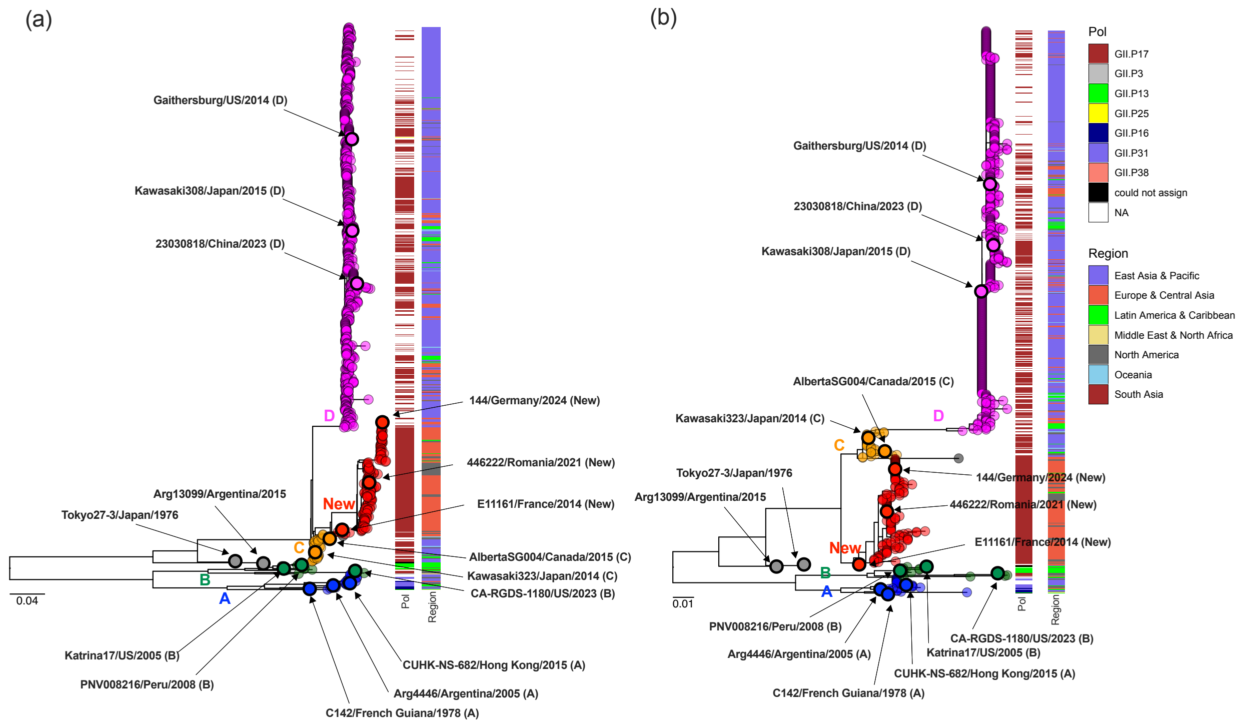

Supplementary Figure 1. Phylogenetic trees generated using (a) VP1-encoding nucleotide and (b) VP1 amino acid sequences with viruses used to develop VLPs indicated. The maximum-likelihood trees were generated using n = 1,013 GII.17 norovirus sequences. Viruses from each cluster selected for VLPs development are indicated by arrows.

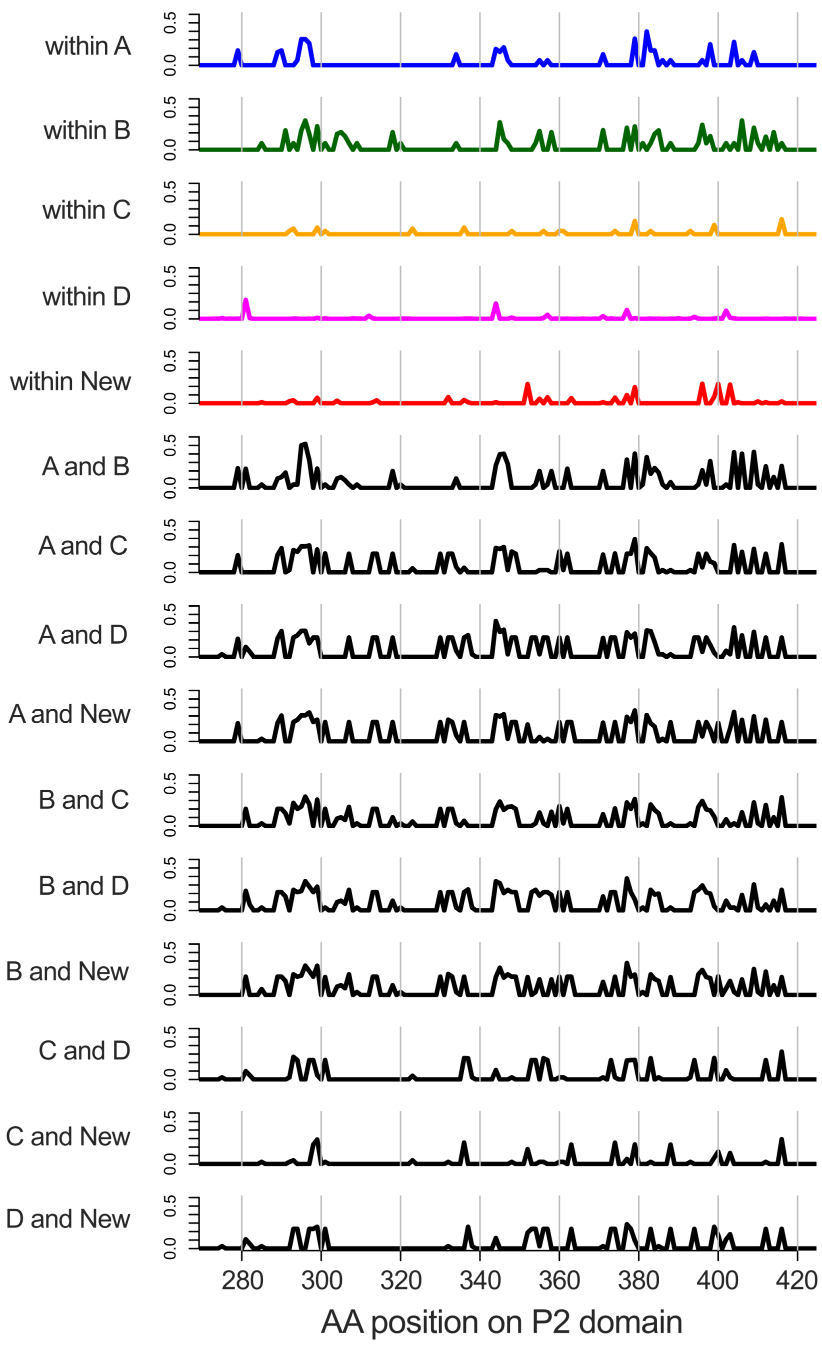

Supplementary Figure 2. Shannon entropy values within P2 subdomain of VP1. The stacked line graphs summarize normalized Shannon entropy values within the P2 subdomain at intra- and inter-cluster levels. To reduce sampling bias, sequences from cluster D and new GII.17 lineage viruses were randomly subsampled to reduce sample size of 777 and 155, respectively, to 30 sequences. Dataset for cluster A, B, and C include 23, 16, and 39 sequences, respectively. Source data is provided on figshare (doi https://doi.org/10.6084/m9.figshare.29421056).

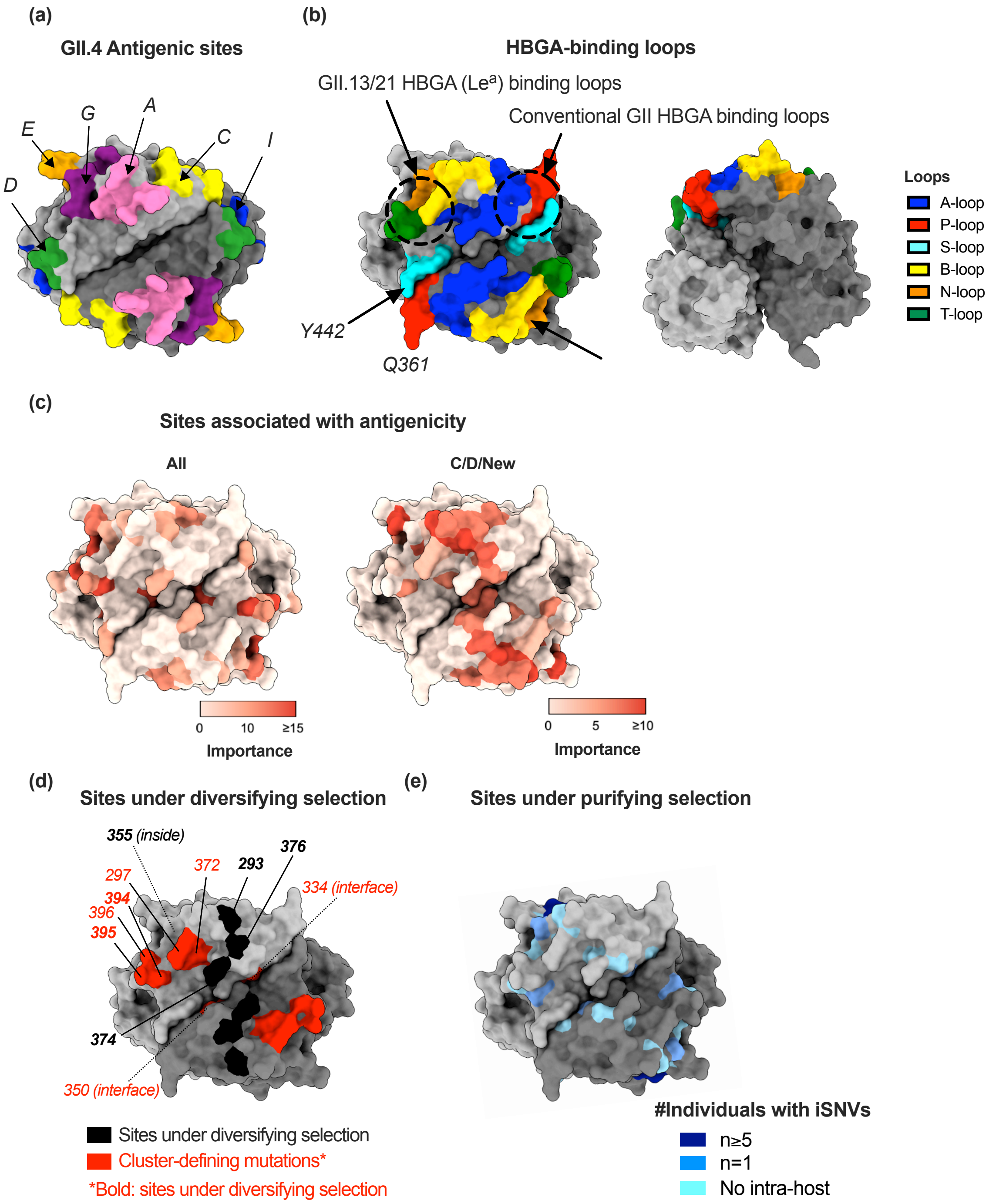

Supplementary Figure 3. Structural mapping of key residues involved in the genetic diversification of human noroviruses. **(a)** Previously characterized major antigenic sites of GII.4 norovirus (PDB: 4OP7), **(b)** HBGA-binding loops and key mutations involved in HBGA-binding of GII.17 norovirus, **(c)** Machine learning-predicted residues important to define antigenic differences among GII.17 noroviruses, **(d)** Residues under diversifying pressure and/or GII.17 cluster-defining mutations detected in Germany surveillance data, and **(e)** Residues under purifying pressure, colored by the patterns of their within-host variation in Germany surveillance data.

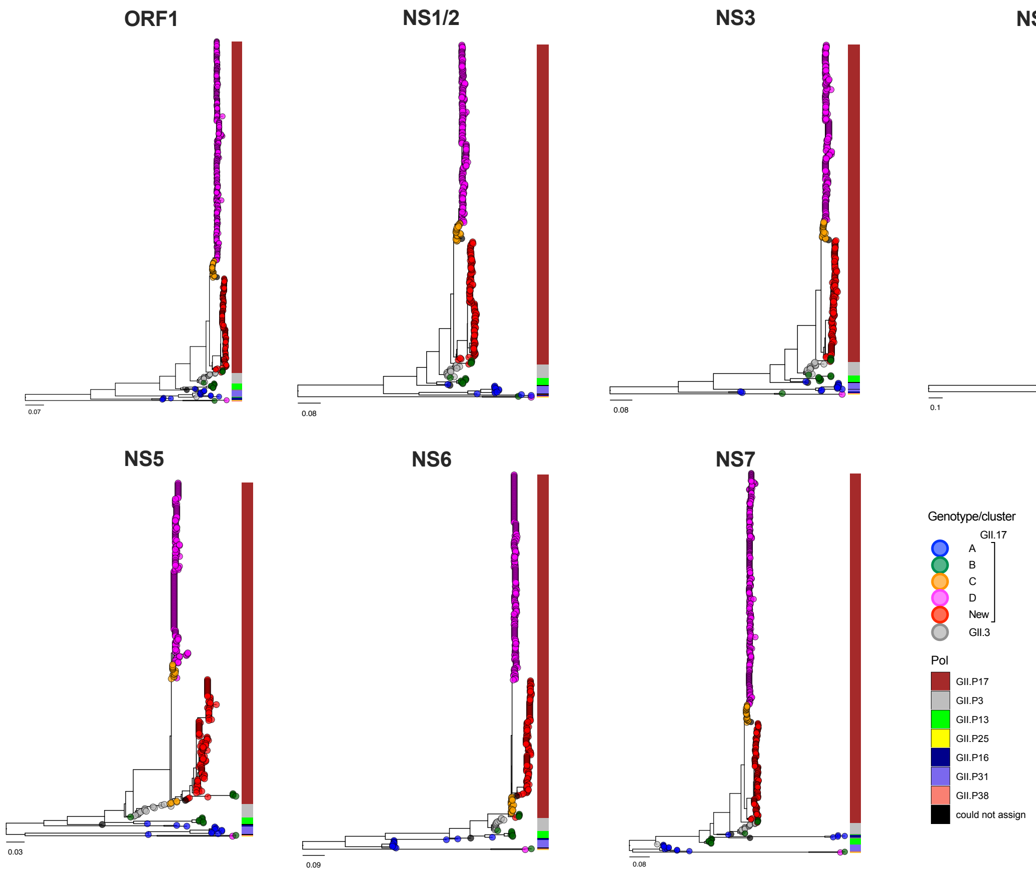

Supplementary Figure 4. Maximum-likelihood trees using ORF1 sequences spanning NS1/2 to NS7 of GII.17 noroviruses. The phylogenetic trees were constructed using sequences from entire ORF1 and individual protein-coding region of each nonstructural protein. The tips on the tree (circles) were color coded by cluster (determined by VP1) following the same color scheme in Figure 1. The heatmap next to the tree indicates corresponding polymerase types.

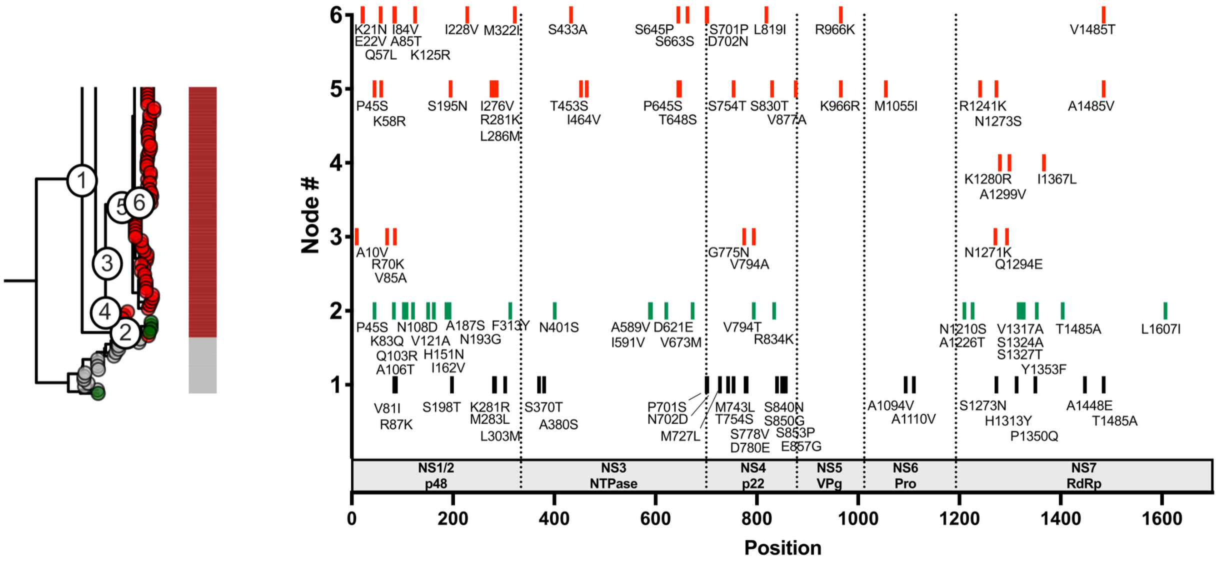

Supplementary Figure 5. Cluster-defining amino acid mutations on the ORF1.

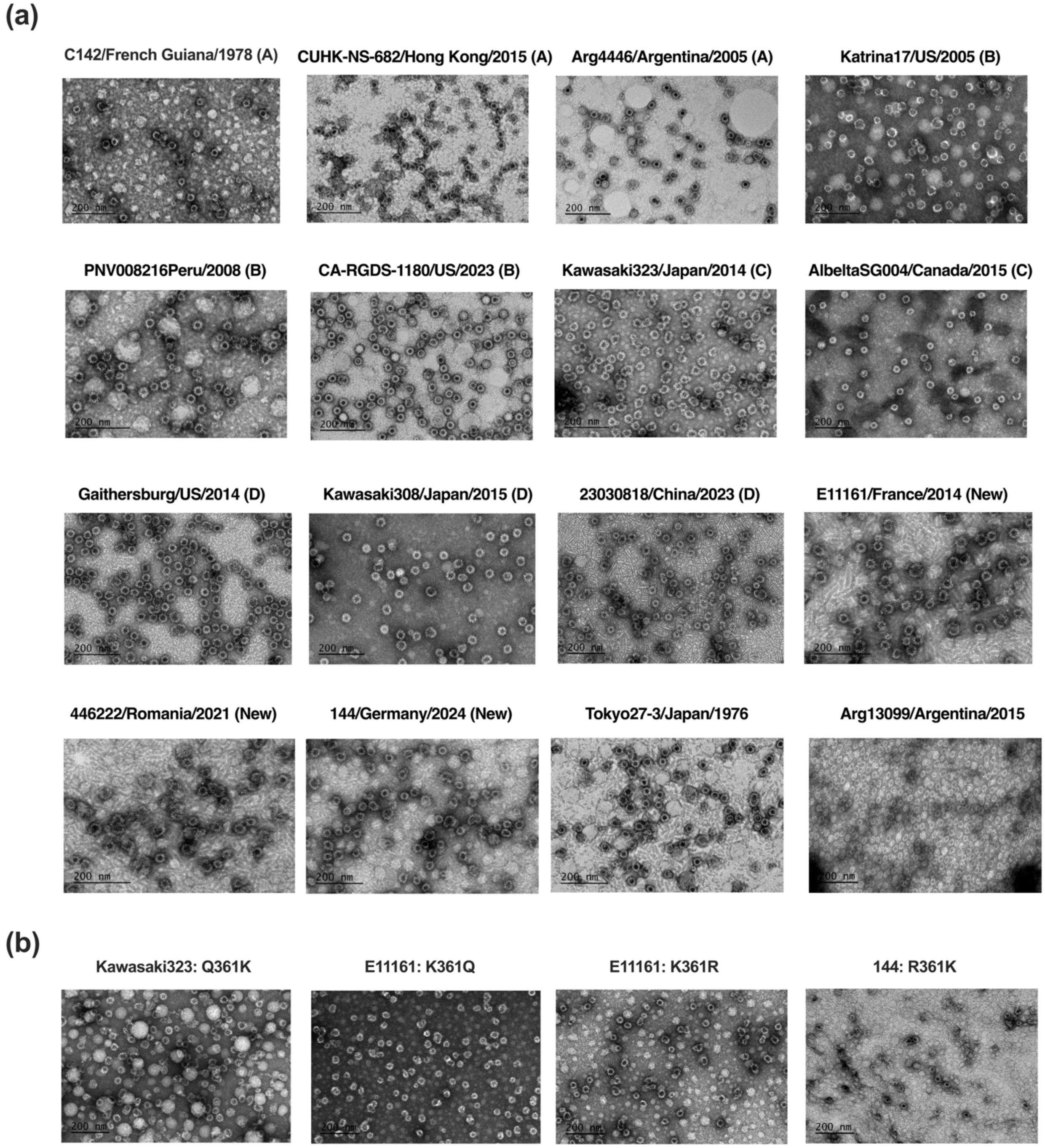

Supplementary Figure 6. Electron microscopy images of VLPs. (a) Wild-type viruses and **(b)** site-directed mutants VLPs.

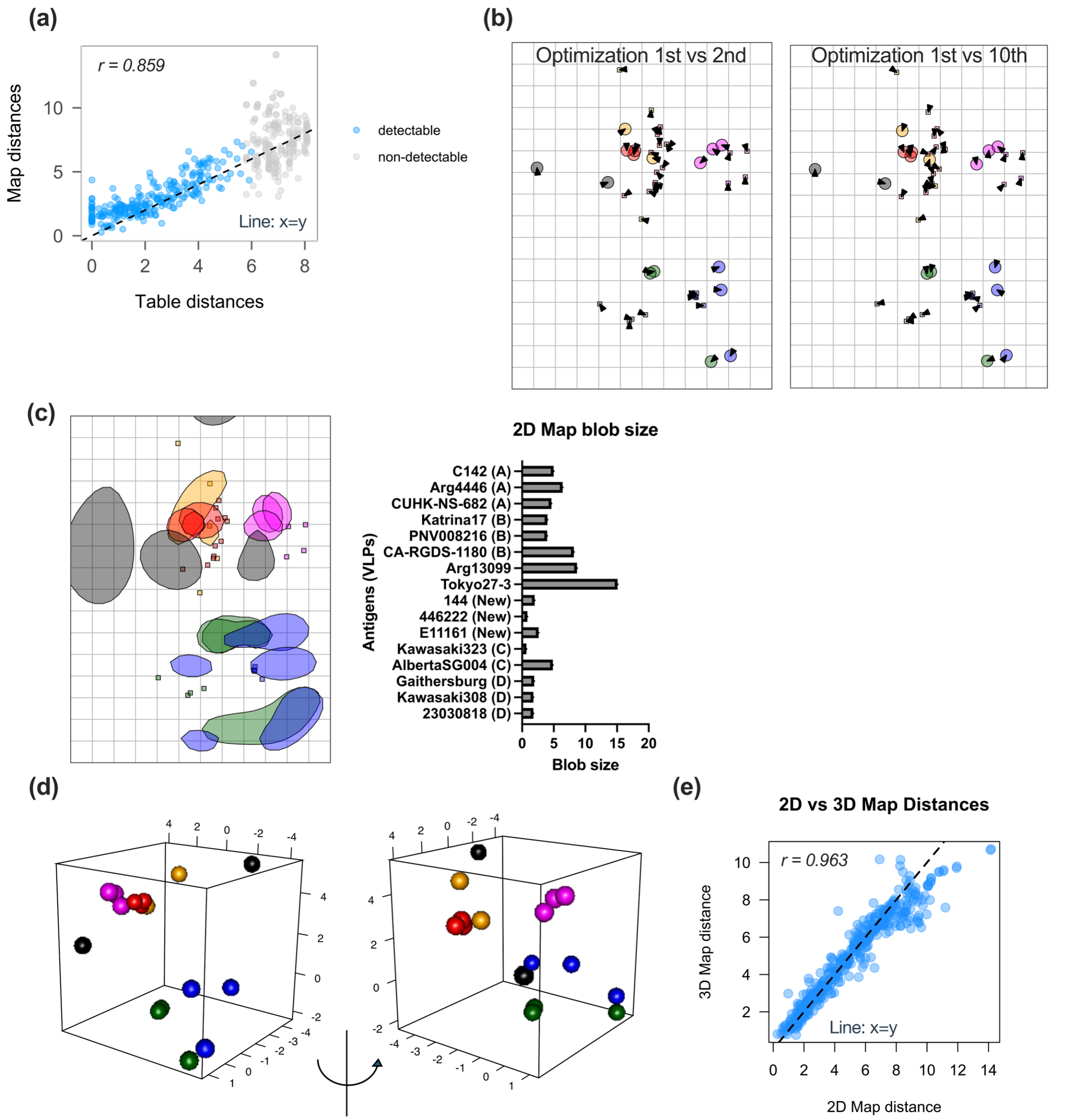

Supplementary Figure 7. Diagnostics of antigenic cartography data. (a) Correlation plot of titer table distance (x axis) and two-dimensional (2D) map distance (y axis) (n =448 data points). **(b)** Convergence after 500 optimizations was confirmed by comparing best and second-best map (left) and 10th-best map (right). The arrows indicate relocation of points during the optimizations. **(c)** Uncertainty of map projection was confirmed by bootstrapping using Bayesian method. The blobs indicate uncertainty of location of antigens (VLPs) and their blob size is summarized in bar graph at the right. **(d)** 3D antigenic map rendered using *rgl* package in R. Only antigens (VLPs) are drawn and indicated by spheres. **(e)** Correlation plot of antigenic distance generated using 2D (x axis) and 3D maps (y axis) (n = 448 data points). Source data is provided on figshare (doi https://doi.org/10.6084/m9.figshare.29421056).

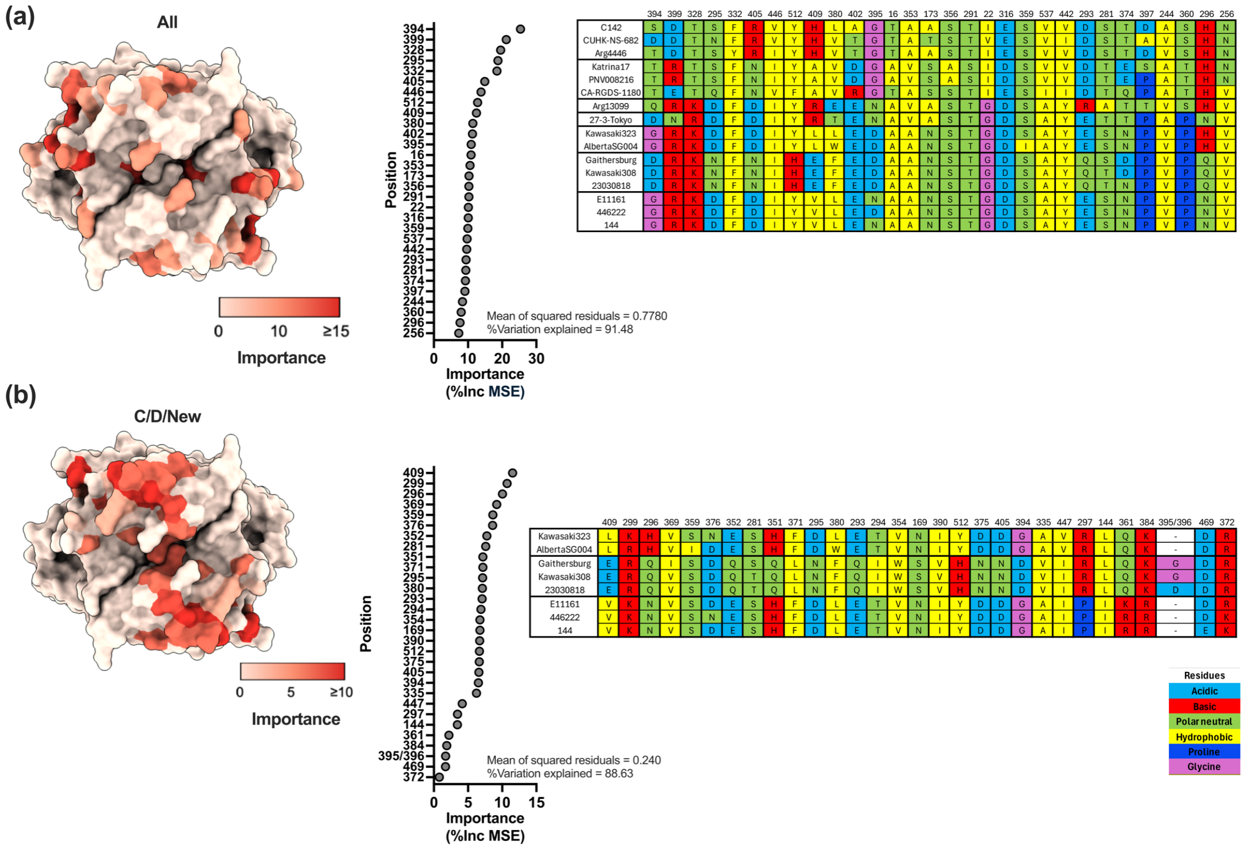

Supplementary Figure 8. Sequence alignments of predicted sites associated with GII.17 antigenic differences. Top 30 important sites were predicted using random forest method and **(a)** the data from all the pairs of viruses and **(b)** the data only including cluster C, D, and new GII.17 noroviruses. The sequence alignments are colored based on the chemical properties of amino acids. The positions of the sites are calculated based on the Kawasaki323/Japan/2014 virus.

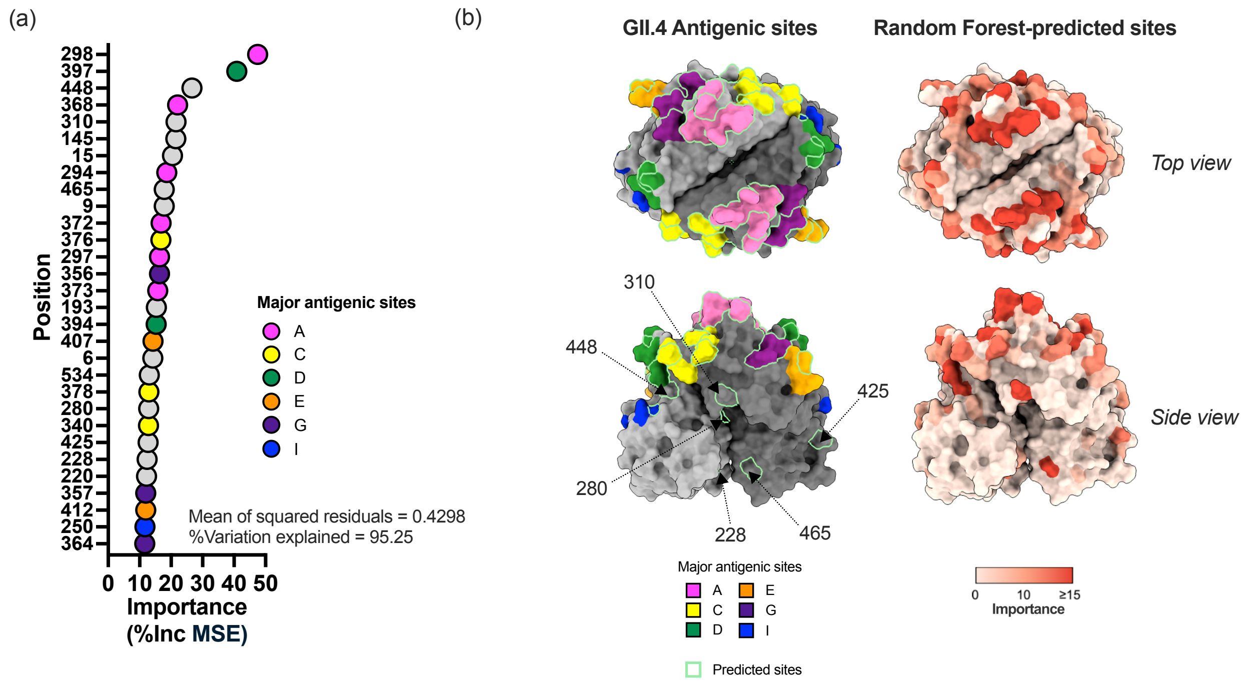

Supplementary Figure 9. Validation analysis of random forest prediction using previously published data of GII.4 noroviruses. (a) Top 30 important sites were predicted using random forest method and the blockade EC_{50} titer tables extracted from a previous study (Kendra et al. Proc Natl Acad Sci U S A. 2021 Mar 16;118(11):e2015874118). The colors of the dot indicate previously characterized major antigenic sites of GII.4 noroviruses. **(b)** Machine learning-predicted residues involved in antigenic differences are highlighted in light green along with previously characterized major antigenic sites of GII.4 norovirus (left; PDB: 4OP7). Residues predicted to be involved in antigenic differences but not mapping to known antigenic sites are indicated by arrows. The color gradient indicates importance of the positions (%Inc MSE; the increase of the mean squared error when given variable is randomly permuted) on the model (right), which are presented in the dot plot in panel a. Source data is provided on figshare (doi https://doi.org/10.6084/m9.figshare.29421056).

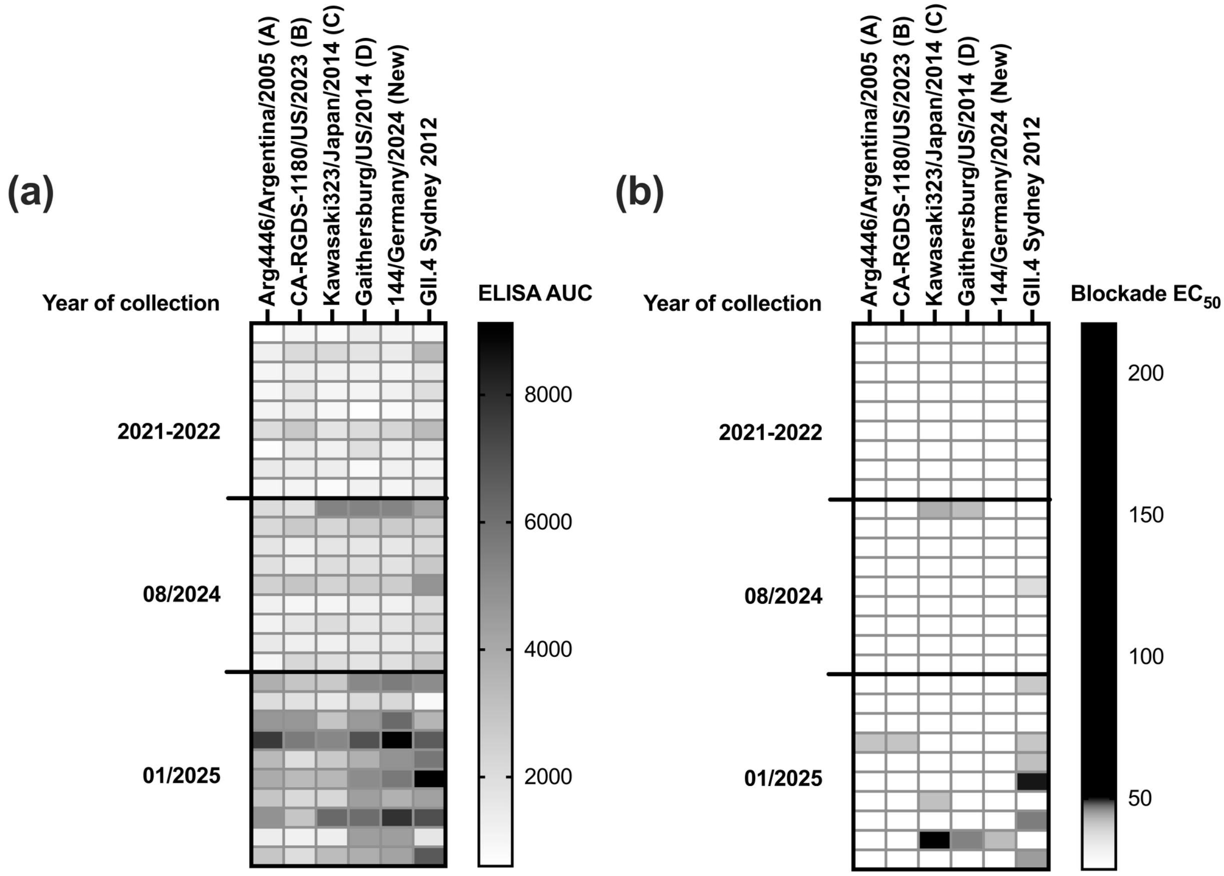

Supplementary Figure 10. Anti-GII.17 antibody titers in individuals. The heatmaps present individual **(a)** ELISA IgG titers and **(b)** HBGA-blockade titers. The columns indicate VLPs tested, and rows indicate individual persons (n = 28). Source data is provided on figshare (doi https://doi.org/10.6084/m9.figshare.29421056).

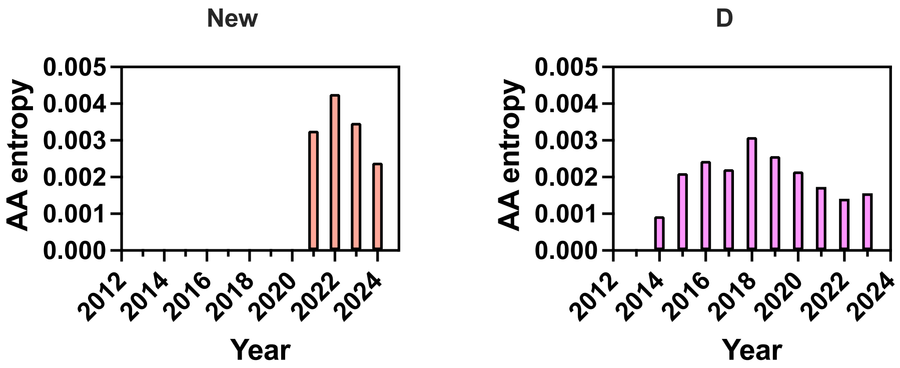

Supplementary Figure 11. Temporal change of Shannon entropy at the global level. Shannon entropy was calculated using global VP1 amino acid sequence dataset (n = 932; left: new cluster, right: cluster D). Source data is provided on figshare (doi https://doi.org/10.6084/m9.figshare.29421056).

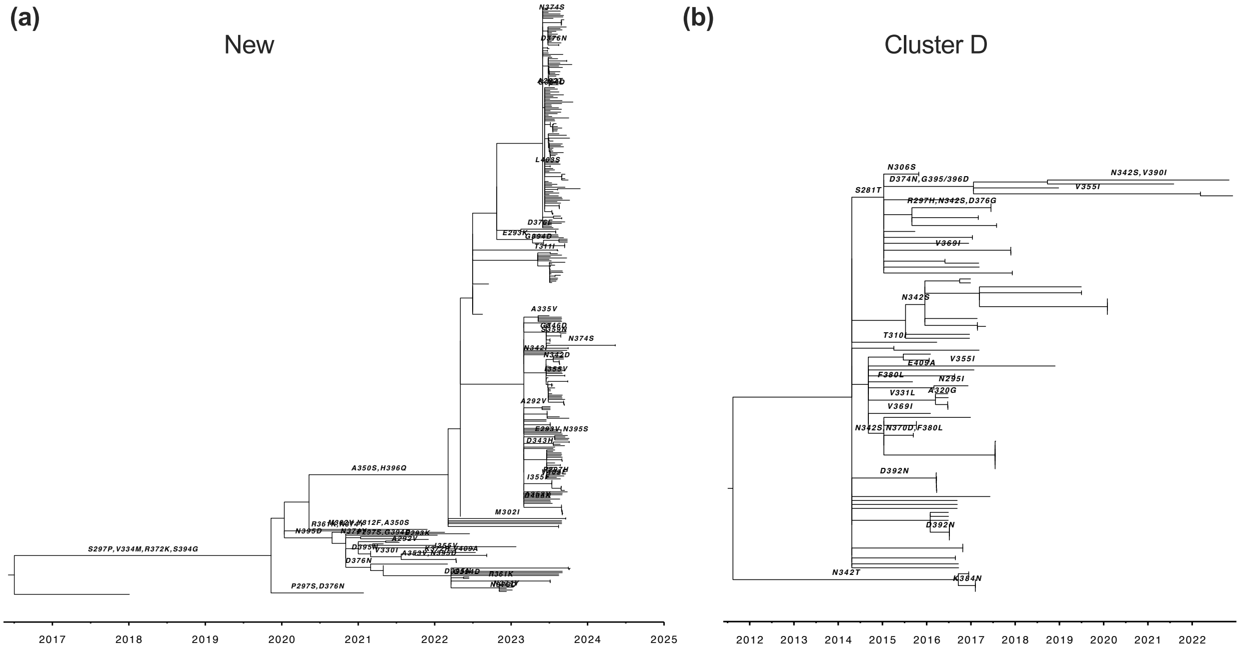

Supplementary Figure 12. Time-calibrated trees and annotated mutations on the nodes detected in Germany surveillance data. Phylogenetic trees of **(a)** new GII.17 noroviruses and **(b)** cluster D viruses detected in Germany since 2015 to 2024. Mutations were mapped on the branches.

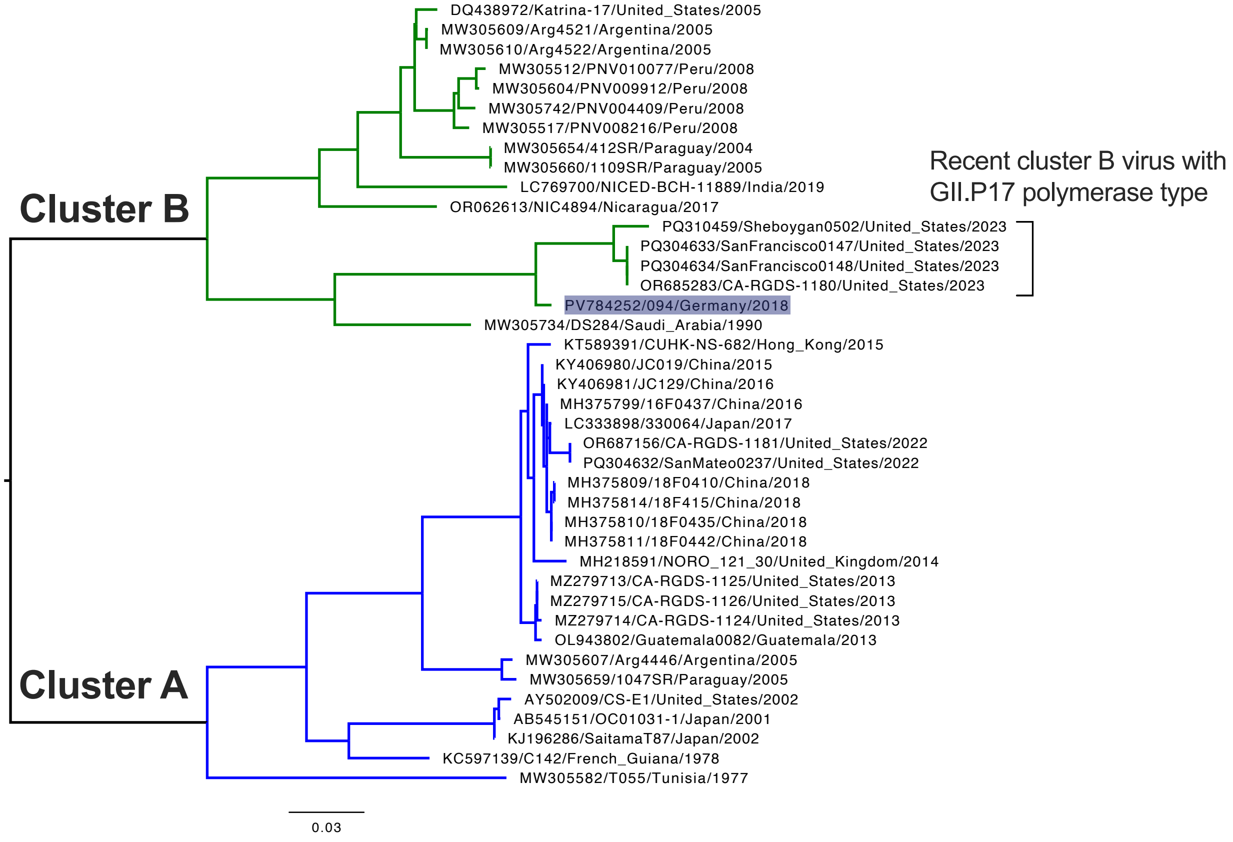

Supplementary Figure 13. Maximum-likelihood tree using VP1-encoding sequences from clusters A and B including a P2 subdomain sequence of cluster B virus detected in Germany. The branches were color coded by cluster. A virus detected in Germany in 2018 that is genetically close to the recent cluster B viruses with GII.P17 polymerase is highlighted on the tree.

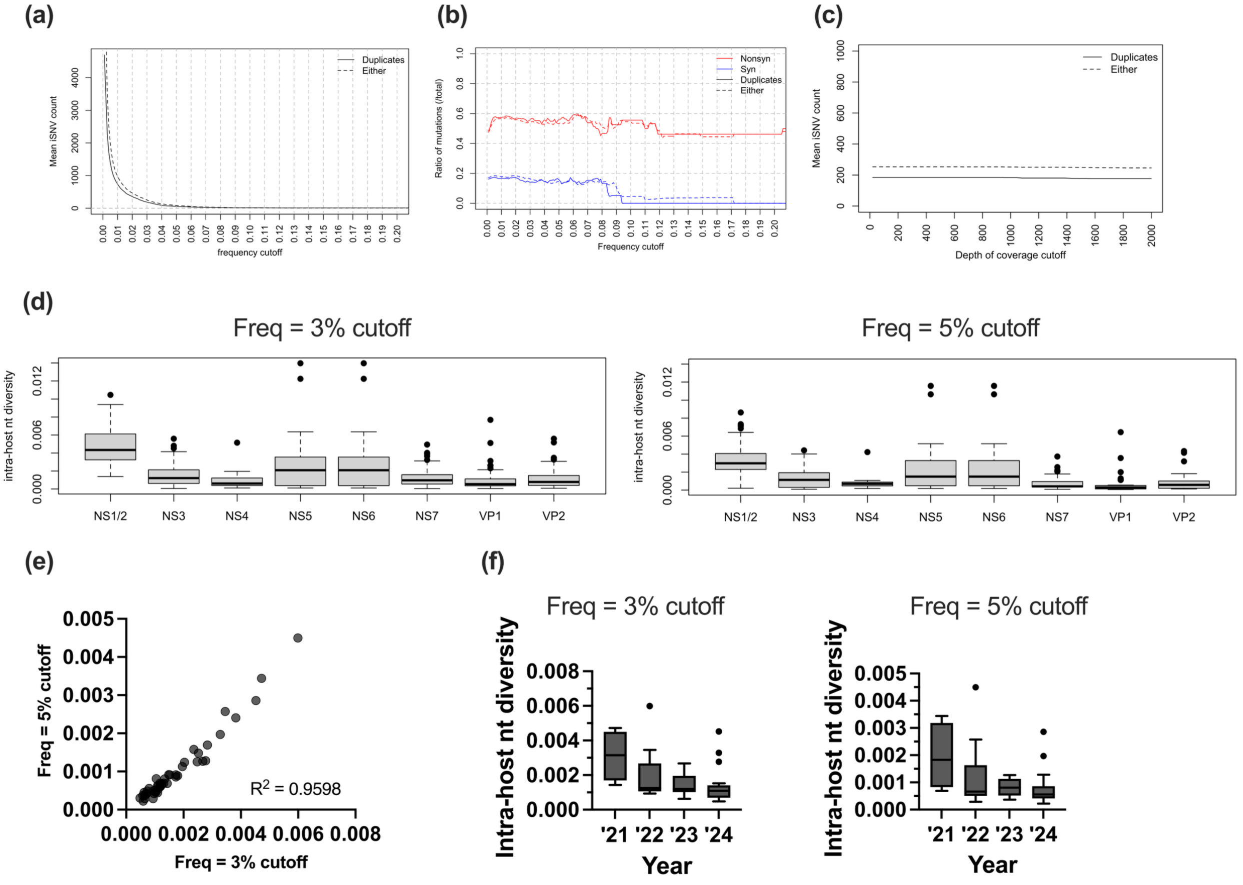

Supplementary Figure 14. Sensitivity analysis of iSNV profiling. (a) Mean number of intra-host single nucleotide variants (iSNVs) detected and **(b)** ratio of nonsynonymous and synonymous iSNVs when using different iSNV frequency cutoff filters. **(c)** Mean number of iSNVs when applying different depth of coverage cutoff filters. Those detected in duplicate runs are indicated by solid line and those detected in either of the duplicate runs are indicated by dashed line. **(d)** Intra-host nucleotide diversity calculated for each protein from viruses detected in 49 individuals using iSNV frequency = 3% (left) and 5% (right) cutoff filters. The boxes indicate the median and 25–75th percentiles, and the whiskers indicate the 1.5 × interquartile ranges from the boxes. **(e)** Correlation plot of mean intra-host nucleotide diversity in 49 individuals calculated using iSNV frequency cutoff = 3% (x axis) and 5% (y axis). **(f)** Temporal trend of intra-host nucleotide diversity when using different iSNV frequency cutoff filters, 3% (left) and 5% (right). The boxplots show the median and 25th–75th percentiles with whiskers created using Tukey's method. Source data is provided on figshare (doi https://doi.org/10.6084/m9.figshare.29421056).

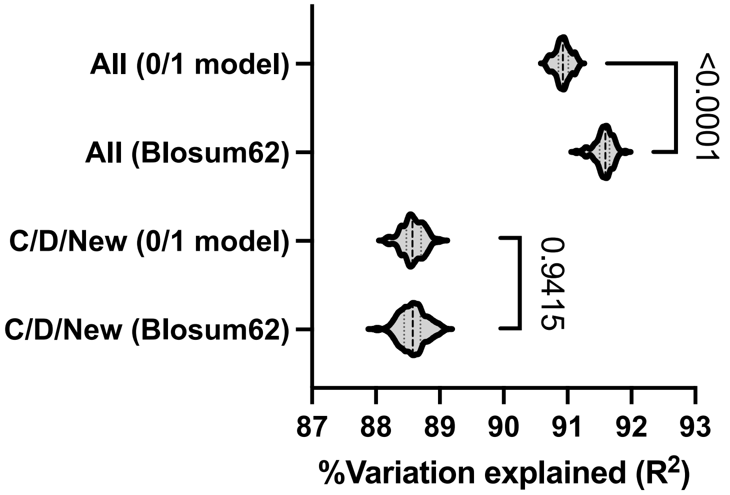

Supplementary Figure 15. Percent variation explained in 100 replicates of random forest predictions using different methods of genetic distance calculation. The sites associated with antigenic differences were predicted using random forest methods using antigenic distance and genetic distance. Here, two methods of genetic distance calculation were used and their performance were compared by assessing %variation explained through 100 replications of runs; (i) the number of changes between the pair, which is 0 for same residues and 1 for different residues used, and (ii) genetic distance calculated using Blosum62 substitution model to generate more continuous values of differences based on the physicochemical similarity of amino acid types used. The violin plot shows the median and quartiles values with the kernel density. P values were provided in t-test (two-sided). Source data is provided on figshare (doi https://doi.org/10.6084/m9.figshare.29421056).

Supplementary Table 1. Viruses used in the immunoassays.

Virus	GenBank accession number	Year	Location	Cluster
23030818	PP267298	2023	China	D
Kawasaki308	LC037415	2015	Japan	D
Gaithersburg	KR083017	2014	United States	D
AlbertaSG004	KX171415	2015	Canada	C
Kawasaki323	AB983218	2014	Japan	C
E11161	KU587625	2014	France	New (2014-2016 sub-cluster)
446222	OP805362	2021	Romania	New (2021-2024 sub-cluster)
144	PV784303	2024	Germany	New (2023-2024 sub-cluster)
Tokyo27-3	AB684681	1976	Japan	–
Arg13099	MW305625	2015	Argentina	–
CA-RGDS-1180	OR685283	2023	United States	B
PNV008216	MW305517	2008	Peru	B
Katrina17	DQ438972	2005	United States	B
CUHK-NS-682	KT589391	2015	Hong Kong	A
Arg4446	MW305607	2005	Argentina	A
C142	KC597139	1978	French Guiana	A

Supplementary Data 1. GenBank accession numbers of GII.17 sequences obtained as part of this study. Provided in an excel file.

Supplementary Data 2. GenBank accession numbers of GII.17 and related norovirus sequences retrieved from GenBank (collected on October 18, 2024). Provided in an excel file.

Supplementary Data 3. Sequence alignment of HBGA-binding loops for GII.17 viruses used for immunoassays. Provided in an excel file.

Supplementary Data 4. Sites under diversifying or purifying selection in P2 subdomain. Provided in an excel file.

Supplementary Data 5. SRA accession numbers of GII.17 sequence fastq files obtained in this study. Provided in an excel file.

Supplementary Data 6. GenBank accession numbers of nucleotide sequences that were used to design customized baits for hybrid capture during next-generation sequencing. Provided in an excel file.
