# Supplementary material for: GII.17 norovirus re-emerged in the 2020s as a result of dynamic and adaptive evolutionary processes
Source: Nat Commun. 2025 Nov 24;16:11596. doi: 10.1038/s41467-025-66279-6 (PMC12749941; doi:10.1038/s41467-025-66279-6)
Supplement: Supplementary file 2 — Description of Additional Supplementary Files [file 41467_2025_66279_MOESM2_ESM.pdf]

Description of Additional Supplementary Files

Supplementary Data 1. GenBank accession numbers of GII.17 sequences obtained as part of this study

Supplementary Data 2. GenBank accession numbers of GII.17 and related norovirus sequences retrieved from GenBank (collected on October 18, 2024)

Supplementary Data 3. Sequence alignment of HBGA-binding loops for GII.17 viruses used for immunoassays

Supplementary Data 4. Sites under diversifying or purifying selection in P2 subdomain

Supplementary Data 5. SRA accession numbers of GII.17 sequence fastq files obtained in this study

Supplementary Data 6. GenBank accession numbers of nucleotide sequences that were used to design customized baits for hybrid capture during next-generation sequencing
