# Supplementary material for: GII.17 norovirus re-emerged in the 2020s as a result of dynamic and adaptive evolutionary processes
Source: Nat Commun. 2025 Nov 24;16:11596. doi: 10.1038/s41467-025-66279-6 (PMC12749941; doi:10.1038/s41467-025-66279-6)
Supplement: Supplementary file 9 — Reporting Summary [file 41467_2025_66279_MOESM9_ESM.pdf]

nature portfolio

Corresponding author(s): Gabriel I. Parra, Kentaro Tohma

Last updated by author(s): Oct 16, 2025

Reporting Summary

Nature Portfolio wishes to improve the reproducibility of the work that we publish. This form provides structure for consistency and transparency in reporting. For further information on Nature Portfolio policies, see our Editorial Policies and the Editorial Policy Checklist.

Statistics

For all statistical analyses, confirm that the following items are present in the figure legend, table legend, main text, or Methods section.

n/a	Confirmed	
☐	☒	The exact sample size (*n*) for each experimental group/condition, given as a discrete number and unit of measurement
☐	☒	A statement on whether measurements were taken from distinct samples or whether the same sample was measured repeatedly
☐	☒	The statistical test(s) used AND whether they are one- or two-sided<br>*Only common tests should be described solely by name; describe more complex techniques in the Methods section.*
☒	☐	A description of all covariates tested
☐	☒	A description of any assumptions or corrections, such as tests of normality and adjustment for multiple comparisons
☐	☒	A full description of the statistical parameters including central tendency (e.g. means) or other basic estimates (e.g. regression coefficient) AND variation (e.g. standard deviation) or associated estimates of uncertainty (e.g. confidence intervals)
☐	☒	For null hypothesis testing, the test statistic (e.g. *F*, *t*, *r*) with confidence intervals, effect sizes, degrees of freedom and *P* value noted<br>*Give P values as exact values whenever suitable.*
☒	☐	For Bayesian analysis, information on the choice of priors and Markov chain Monte Carlo settings
☒	☐	For hierarchical and complex designs, identification of the appropriate level for tests and full reporting of outcomes
☐	☒	Estimates of effect sizes (e.g. Cohen's *d*, Pearson's *r*), indicating how they were calculated

Our web collection on statistics for biologists contains articles on many of the points above.

Software and code

Policy information about availability of computer code

Data collection	We used HIVE-hexagon aligner v2.1, HIVE-heptagon profiler v2.1, SPAdes v3.14.0, BLAST+ v2.9.0, and Bowtie2 v2.4.4 to obtain and analyze viral sequence data. BWA v0.7.19-r1273, Samtools v1.22, and seqtk v1.5 were used to remove human-detived sequences from the NGS fastq files. Intra-host single nucleotide variants data were collected using HIVE-heptagon profiler v2.1.
Data analysis	MEGA v11, MAFFT v7 (online version), IQ-TREE multicore version 1.6.12 (online version), and TreeTime v0.11.4 were used to conduct genetic and phylogenetic analyses. R v4.5.0 with ggtree package v3.16.0 and FigTree v1.4.4 were used to visualize the phylogenetic trees. R v4.5.0 with phangorn v2.12.1 and HDMD v1.2 were used to analyze amino acid sequences. Selective pressure analyses were conducted using Datamonkey Adaptive Evolution Server. Structural data was visualized using USCF ChimeraX v1.9. The structural model was predicted using AlphaFold v2 implemented in FDA HIVE platform. Antigenic cartography analyses were conducted using R v4.5.0 and Racmacs package v1.2.9. Correlation analysis was conducted using R v4.5.0 and stats package v4.5.1. Random Forest regression analyses were conducted using R v4.5.0 and randomForest package v4.7-1.2. Statistical analyses and data visualization were performed using R v4.5.0 with graphics package v4.5.1 and GraphPad Prism v10. Geographic map was created using R v4.5.0 with sf v1.0-21 and spData v2.3.4 packages.

For manuscripts utilizing custom algorithms or software that are central to the research but not yet described in published literature, software must be made available to editors and reviewers. We strongly encourage code deposition in a community repository (e.g. GitHub). See the Nature Portfolio guidelines for submitting code & software for further information.

Data

Policy information about availability of data

All manuscripts must include a data availability statement. This statement should provide the following information, where applicable:

- Accession codes, unique identifiers, or web links for publicly available datasets
- A description of any restrictions on data availability
- For clinical datasets or third party data, please ensure that the statement adheres to our policy

The generated fasta files and fastq files obtained in this study were deposited in the GenBank and SRA (BioProject: PRJNA1279050), respectively. Accession numbers are listed in Supplementary Tables 1 and 6. All sequences deposited in GenBank will be released upon acceptance of the manuscript.

Source data obtained in evolutionary analyses, immunoassays (ELISA titers), qPCR (viral genome titers), #outbreaks caused by norovirus GII in the United States (obtained at CaliciNet website), and the number of sequences associated with norovirus GII.17 deposited in GenBank were deposited at FigShare (DOI: https://doi.org/10.6084/m9.figshare.29421056).

Research involving human participants, their data, or biological material

Policy information about studies with human participants or human data. See also policy information about sex, gender (identity/presentation), and sexual orientation and race, ethnicity and racism.

Reporting on sex and gender	This information has not been collected.
Reporting on race, ethnicity, or other socially relevant groupings	This information has not been collected.
Population characteristics	This information has not been collected.
Recruitment	No human participants were recruited in this study.
Ethics oversight	Human fecal samples were collected as part of national disease surveillance program. All data was de-identified and ethical approval was not required. Commercially available human serum and saliva samples were purchased from a company. Donated human serum samples were obtained from NIH blood bank. Additional human saliva samples were collected from volunteers under the IRB protocol number: CBER IRB 16-069B and NIAID IRB 11-I-0109. All these samples were de-identified.

Note that full information on the approval of the study protocol must also be provided in the manuscript.

Field-specific reporting

Please select the one below that is the best fit for your research. If you are not sure, read the appropriate sections before making your selection.

☐ Life sciences ☐ Behavioural & social sciences ☒ Ecological, evolutionary & environmental sciences

For a reference copy of the document with all sections, see nature.com/documents/nr-reporting-summary-flat.pdf

Ecological, evolutionary & environmental sciences study design

All studies must disclose on these points even when the disclosure is negative.

Study description	In this study, we sequenced genomes of noroviruses collected from patients with acute gastroenteritis as part of national acute gastroenteritis surveillance programs from Germany, Spain, and Argentina. We conducted genomics and evolutionary analyses of newly obtained and publicly available viral sequences. In addition, we expressed viral proteins and conducted immunoassays using human and animal samples to determine phenotypic characteristics of noroviruses obtained in this study.
Research sample	Human fecal samples were collected from patients with acute gastroenteritis and sent for molecular testing at national reference laborotires. Norovirus GII.17-positive fecal samples were selected for further characterization and full-length viral sequencing in this study. Human serum and saliva samples were obtained from a company or NIH Blood Bank, or collected as part of this study to conduct phenotypic characterization of noroviruses identified in this and previous studies. Animal (mouse and guinea pig) serum samples were collected from animals immunized with norovirus proteins to conduct immunoassays.
Sampling strategy	No sample size calculation was performed for human samples, which were collected from patients presenting acute gastroenteritis as part of national surveillance programs. Human saliva and serum samples were not subjected to sample size calculation as those samples were used for descriptive phenotypic characterization of viruses. Mouse serum samples were collected from 5 mice per group (including attrition) to conduct ELISA-based immunoassays. The statistical significance of the number of animals per group was calculated using a level of significance of 5% and a study power of 80% according to Charan and Kantharia, How to calculate sample size in animal studies? J Pharmacol Pharmacother. 2013 Oct-Dec; 4(4): 303–306. Guinea pig serum samples were collected from 2 guinea pigs per group and used as secondary detection antibodies of norovirus proteins in ELISA-based assays.

Data collection	Human fecal samples and surveillance data were collected by local public health authorities, diagnostic laboratories, or physicians and de-identified by Department of Infectious Diseases, Robert Koch Institute, Berlin, Germany; Enterovirus and Viral Gastroenteritis Unit, National Centre for Microbiology, Instituto de Salud Carlos III, Madrid, Spain; and Laboratory of Viral Gastroenteritis, INEI-ANLIS "Dr. Carlos G. Malbrán", Buenos Aires, Argentina, for further analyses.
Timing and spatial scale	The data was collected as part of routine national surveillance programs of Germany, Spain, and Argentina. We used data of norovirus GII.17 positive cases, which were obtained during recent outbreaks of norovirus in these countries mainly during 2021-2024.
Data exclusions	No data was excluded.
Reproducibility	Site-directed mutagenesis assays were repeated in different days and confirmed the reproducibility. The large-scale ELISA assays were conducted in duplicate wells and if the data presented variability, the experiment was repeated to obtain reproducible data.
Randomization	The data is collected as part of disease surveillance and no randomization is performed in this study.
Blinding	All human samples were de-identified in this study.

Did the study involve field work? ☐ Yes ☒ No

Reporting for specific materials, systems and methods

We require information from authors about some types of materials, experimental systems and methods used in many studies. Here, indicate whether each material, system or method listed is relevant to your study. If you are not sure if a list item applies to your research, read the appropriate section before selecting a response.

Materials & experimental systems

n/a	Involved in the study	
☐	☒	Antibodies
☒	☐	Eukaryotic cell lines
☒	☐	Palaeontology and archaeology
☐	☒	Animals and other organisms
☒	☐	Clinical data
☒	☐	Dual use research of concern
☒	☐	Plants

Methods

n/a	Involved in the study	
☒	☐	ChIP-seq
☒	☐	Flow cytometry
☒	☐	MRI-based neuroimaging

Antibodies

Antibodies used

Anti-norovirus guinea pig serum (Ford-Siltz et al. J Infect Dis, 2020, 1:5000 dilution); Goat Anti-Guinea Pig IgG (H + L) antibody, peroxidase-labeled (SeraCare, Cat#5220-0366, 1:2000 dilution); Anti-norovirus cross-reactive mouse monoclonal antibody 30A11 (Tohma et al. mBio, 2019, 1:10000 dilution); Monoclonal Anti-Blood Group Lewis a mouse antibody (Sigma-Aldrich, Cat#SAB4700762, clone 7LE, 1:100 dilution); Monoclonal Anti-Blood Group Lewis b mouse antibody (abcam, Cat#AB3968, clone 2-25LE, 1:100 dilution); Anti-Lewis x monoclonal mouse antibody (Calbiochem, Cat# 434631, clone P12, 1:100 dilution); Anti-Lewis y monoclonal mouse antibody (Calbiochem, Cat#434636, clone F3, 1:100 dilution); Blood Group Antigen H (O) Type 1 mouse monoclonal Antibody (Invitrogen, Cat#14-9810-82, clone 17-206, 1:100 dilution); Blood Group H Type 2 mouse monoclonal Antibody (Invitrogen, Cat#MA1-35386, clone 19-OLE, 1:100 dilution); Goat anti-mouse IgG antibody, peroxidase-labeled (SeraCare, Cat#5220-0339, 1:2000 dilution); goat anti-mouse IgA + IgG + IgM antibody, peroxidase-labeled (SeraCare, Cat#5220-0342, 1:2000 dilution); Goat anti-human IgG antibody, peroxidase-labeled (SeraCare, Cat#5220-0330, 1:2000 dilution).

Validation

Anti-norovirus cross-reactive mouse monoclonal antibody 30A11 was produced and characterized in previous studies (Tohma et al. mBio, 2019; Ford-Siltz et al. Microbiol Spectr. 2024). Other primary antibodies were purchased from Sigma-Aldrich, abcam, Calbiochem, and Invitrogen.

Monoclonal Anti-Blood Group Lewis a mouse antibody (Sigma-Aldrich, Cat#SAB4700762, clone 7LE): https://www.sigmaaldrich.com/US/en/product/sigma/sab4700762?srsltid=AfmBOopcfyYTuWtY10jlryxSfzlOCjQp74bEJQHzREz-ok3LwJdxPFxM

Monoclonal Anti-Blood Group Lewis b mouse antibody (abcam, Cat#AB3968, clone 2-25LE): https://www.abcam.com/en-us/products/primary-antibodies/blood-group-lewis-b-antibody-2-25le-ab3968?srsltid=AfmBOoo2rnD5pJuLmzua-FewWNstfMn4FUNmRh_T87J4iq9Ms44aS8CX

Anti-Lewis x monoclonal mouse antibody (Calbiochem, Cat# 434631, clone P12): https://www.sigmaaldrich.com/US/en/product/mm/434631?srsltid=AfmBOooNfXgLNpHliCceNZGiXSIsyn4JLi7-8_bStsjQuHOFyPJ36CxC

Anti-Lewis y monoclonal mouse antibody (Calbiochem, Cat#434636, clone F3: https://www.sigmaaldrich.com/US/en/product/mm/434636

Blood Group Antigen H (O) Type 1 mouse monoclonal Antibody (Invitrogen, Cat#14-9810-82, clone 17-206): https://

www.thermofisher.com/antibody/product/Blood-Group-Antigen-H-O-Type-1-Antibody-clone-17-206-Monoclonal/14-9810-82

Blood Group H Type 2 mouse monoclonal Antibody (Invitrogen, Cat#MA1-35386, clone 19-OLE, 1:100 dilution): https://www.thermofisher.com/antibody/product/Blood-Group-H-Type-2-Antibody-clone-19-OLE-Monoclonal/MA1-35386

Animals and other research organisms

Policy information about studies involving animals; ARRIVE guidelines recommended for reporting animal research, and Sex and Gender in Research

Laboratory animals	Female BALB/cAnNCr mice (Charles River Laboratories, strain code #555) with five to six weeks of age. Adult female guinea pigs (Charles River Laboratories, strain Hartly) with a weight of 501-550 g at the beginning of the immunization (average 8–12 weeks of age based on the growth chart available at Charles River Laboratories).
Wild animals	The study did not involve wild animals.
Reporting on sex	Animals were used to collect hyperimmune sera to conduct immunoassays and no sex-based analyses using animals were performed.
Field-collected samples	The study did not involve field-collected samples.
Ethics oversight	Animal protocol to collect mouse serum was approved by the FDA Institutional Animal Care and Use Committee (IACUC) (protocol number 2018-41). Animal protocol to collect guinea pig serum was approved by the FDA IACUC (protocol number 2017–29).

Note that full information on the approval of the study protocol must also be provided in the manuscript.

Plants

Seed stocks	*Report on the source of all seed stocks or other plant material used. If applicable, state the seed stock centre and catalogue number. If plant specimens were collected from the field, describe the collection location, date and sampling procedures.*
Novel plant genotypes	*Describe the methods by which all novel plant genotypes were produced. This includes those generated by transgenic approaches, gene editing, chemical/radiation-based mutagenesis and hybridization. For transgenic lines, describe the transformation method, the number of independent lines analyzed and the generation upon which experiments were performed. For gene-edited lines, describe the editor used, the endogenous sequence targeted for editing, the targeting guide RNA sequence (if applicable) and how the editor was applied.*
Authentication	*Describe any authentication procedures for each seed stock used or novel genotype generated. Describe any experiments used to assess the effect of a mutation and, where applicable, how potential secondary effects (e.g. second site T-DNA insertions, mosiacism, off-target gene editing) were examined.*
